# Bibliographical

**Published:** 1856-07

**Authors:** 


### EDITORIAL DEPARTMENT.

### BIBLIOGRAPHICAL.

*The Louisville Review, a bi-monthly Journal of Practical Medicine and Surgery.* Edited by S. D. Gross, M. D., and T. G. Richardson, M. D.

This is a new candidate for the favor of the medical profession, and judging by the present number, likely to be successful. The names alone of the editors are sufficient to ensure for it a respectful and cordial reception.

We notice particularly a biography of the late Dr. Daniel Drake, by Dr. Yandell, and a sketch of the eminent German surgeon, Richter, and his cotemporaries, by Dr. Gross. In the course of this article, Dr. G. undertakes the defence of Benjamin Bell, against whom every student of medicine has been prejudiced by the dashing philippics of that brilliant but eccentric genius John Bell. Dr. Gross is very severe upon John, whom, with all his talents, he regards as little better than a charlatan.

*History of the Ligature applied to the Brachio-cepholic Artery;* with Statistics of the Operation. By Paul F. Eve, M. D., Nashville. 1856.

In this paper, read before the Tennessee State Medical Society, Dr. Eve has given the statistics of sixteen attempts to tie the *arteria innominata.* In only thirteen was the ligature actually applied and these were all fatal. It is but justice to say that this is the largest number of these operations ever collected in one list, Erichsen enumerating only nine and Velpeau ten. Dr. Eve attributes the ill success of the operation to the impossibility of obliterating the subclavian. He is decidedly opposed to attempting to tie this great trunk.

*The American Journal of Science and Arts, for July.*

Contents: Notice of microscopic forms found in the sea of Kamschatka, with a plate, by Prof. J. W. Bailey; examination of two sugars from California, by S. W. Johnson; composition of muscles in the animal system, by Valenciennes & Fremy; review of the classification of crustacea, by J. D. Dana; on the mode of testing building materials, by Professor Henry; iron ores in the Azoic system, by J. D. Whitney; obituary of Prof. Zadoc Thompson; influence of solar radiation on the vital powers of plants, by J. H. Gladstone; reports of explorations and survey for Pacific railroad; five new mineral species, by Prof. Charles U. Shepherd; correspondence of M. Jerome Niclés; Scientific Intelligence.

*The Mutual Responsibilities of Physicians and the Communiiy.* By Henry P. Tappau, D. D., LL. D.

Dr. Tappan is chancellor of the University of Michigan, and delivered this address as a valedictory to the last graduating class. It is chiefly a defence of medical schools in general and of the University of Michigan in particular. There is of course the usual glorification of doctors common on such occasions, the only times at which the hard-worked practitioner sees much in his fate wherein to glory.

